# Deciphering isonicotinylation and mast cell-associated pathogenic patterns in Duchenne muscular dystrophy: evidence from ensemble learning-driven multi-omics and *in vitro* validation

**DOI:** 10.3389/fmolb.2026.1853169

**Published:** 2026-10-13

**Authors:** Sunrui Yu, Duojia Xu, Yuting Hua, Hangfeng Mo, Yujin Liu

**Affiliations:** 1 Department of Anesthesiology, Jinhua Municipal Central Hospital, Jinhua, Zhejiang, China; 2 Nursing Department, Jiangyin People’s Hospital, Jiangyin, China; 3 Nursing Department, the First People’s Hospital of Wenling, Wenling, Zhejiang, China; 4 Nursing Department, the Second Hospital of Jinhua, Jinhua, Zhejiang, China

**Keywords:** Duchenne muscular dystrophy, ensemble learning, isonicotinylation, mast cell, multi-omics

## Abstract

**Background:**

Duchenne muscular dystrophy (DMD) is dystrophin-deficiency skeletal and cardiac muscle disease. Protein post-translational modification (PTM) and aberrant immune cell activation play a key role in its pathogenesis.

**Objective:**

Our study aims to investigate isonicotinylation (Kinic)-associated signature modulatory patterns in mast cells and DMD pathogenesis.

**Methods:**

We first performed integrated bioinformatic methods, such as WGCNA, GSVA, and CIBERSORT to identify Kinic and mast cell (KM)-related differentially expressed genes (DEGs) in DMD patient muscle bulk profiles (GSE38417 and GSE109178). Next, consensus clustering and assembling machine learning pipeline identified KM-associated diagnostic model and molecular subgroups in training the DMD patient muscle bulk set (GSE6011), and then, diagnostic model performance was examined in the independent DMD patient muscle bulk set (GSE32720). In addition, SHAP analysis decoded the main contributor in KM-associated diagnostic model construction, and contributor molecular and immune characters in DMD were estimated in GSE6011 and the DMD mouse muscle single-cell transcriptomic dataset (GSE213925) assisted by computational virtual cells. In addition, the deep learning framework and network pharmacology identified the drug synergy strategy for the treatment of DMD. Finally, the *in vitro* assay identified expression patterns of the main contributor.

**Results:**

KM-associated molecular patterns can potentially guide the diagnostic and molecular stratification for DMD patients. USP18 can be considered an upregulated and mast cell-distributed hub gene involved in pathogenesis of DMD. In addition, finafloxacin with BRD-K10482608 can be considered two potential therapeutic candidates for DMD patient clinical applications.

**Conclusion:**

Our investigation described a novel forecasting and therapeutic model for DMD patients, which potentially enhances DMD patient clinical translation.

## 1 Introduction

Duchenne muscular dystrophy (DMD) is an X-linked dystrophinopathy caused by pathogenic variants in the DMD gene, resulting in dystrophin deficiency and progressive degeneration of skeletal and cardiac muscles (Sun et al., 2020; Darras et al., 1993; Gandhi et al., 2024). Recent studies highlighted that the protein post-translational modification (PTM) in pathogenic proteins can aggravate the progression of DMD (Wei et al., 2024). In addition, dysregulation of immune infiltration in skeletal muscle can lead to chronic inflammation in a DMD mouse model (Hamamura et al., 2024). However, the insights into PTM in regulation of immune cells have not yet been elucidated.

Isonicotinylation (Kinic), a novel PTM, was first reported in liver cancer, modifying histone for increasing oncogenic protein expression and activation of the PI3K–AKT pathway (Jiang et al., 2021). Next, another investigation also identified that Kinic can catalyze non-histone proteins that contribute to the epithelial–mesenchymal transition (EMT) and invasion of liver cells (Li et al., 2024). In addition, an independent study using artificial intelligence (AI) also highlighted predictive potentials of Kinic in risk stratification and drug design of hepatocellular carcinoma (HCC) patients (Zhou et al., 2026). However, Kinic-associated implications in regulation of the immune systems have not yet been clarified. In addition, mast cells, as mediators of inflammation, are also directly linked to DMD pathogenesis (Gorospe et al., 1994). For example, recruitment of mast cells to damaged muscle tissues can lead to progressive myofiber necrosis and induction of inflammation (Huard et al., 2016). However, whether the mast cell balance can be considered an indicator for DMD patients has not yet been elucidated.

According to this, in this study, we pointed out a Kinic and mast cell (KM)-related predictive and multi-target framework for DMD patients and highlighted KM-associated USP18 pathogenic patterns in DMD pathogenesis. In conclusion, our study offers a new insight into the pathogenic mechanisms of DMD patients and provides novel information into DMD patient clinical applications.

## 2 Materials and methods

### 2.1 Bulk profiles and pre-processing

All bulk profiles of DMD muscle tissues were sourced from the GEO database, which was retained using the GEOquery package in R (Davis and Meltzer, 2007). We illustrated dataset information in Table 1. Next, these datasets were normalized for elimination of batch effects using the sva and Limma packages in R for further analysis (Ritchie et al., 2015; Leek et al., 2012). The Kinic-associated gene list was acquired from the published literature, which is listed in Supplementary Table S1.

**TABLE 1:** DMD patient bulk dataset identification.

ID	Samples	Platform	References
GSE38417	6 normal muscle samples and 16 DMD muscle samples	GPL570	Lai and Chen (2021)
GSE109178	6 normal muscle samples and 17 DMD muscle samples	GPL570	Dadgar et al. (2014)
GSE6011	14 normal muscle samples and 23 DMD muscle samples	GPL96	Pescatori et al. (2007)
GSE32720	8 normal muscle samples and 8 DMD muscle samples	GPL14713	Baron et al. (2011)

### 2.2 GSVA and WGCNA analyses

First, in GSE38417, we performed GSVA analysis using R to identify the Kinic patterns in DMD patients compared to normal controls (Hänzelmann et al., 2013). In addition, in GSE109178, we quantified immune features in DMD patients compared to normal controls using the CIBERSORT package in R (Chen et al., 2018). WGCNA in R was performed on GSE38417 and GSE109178 with soft-thresholding powers (β) of 22 and 7, respectively, to achieve a scale-free topology fit. Genes were clustered into modules using dynamic tree cutting with a minimum module size of 60 (20). The module exhibiting the highest positive correlation with the Kinic-related pattern in GSE38417 and GSE109178 was selected (Langfelder and Horvath, 2008). After confirming highest correlated modules related to Kinic in GSE38417 and mast cell in GSE109178, we intersected module genes in these two modules for the identification of KM-related differentially expressed genes (DEGs). The feature importance of these KM-related DEGs was evaluated using the GOSemSim package in R (Yang et al., 2023). Interactions between these DEGs were estimated using the STRING database (Szklarczyk et al., 2023). Moreover, molecular features of these KM-related DEGs were assessed using the KEGG and GO analyses in accordance with the MSIDB-sourced hallmark gene set via the clusterProfiler package in R (Yu et al., 2012).

### 2.3 Consensus clustering

In the GSE6011 DMD patient sample, we performed consensus clustering for the identification of KM-related subgroups based on KM-associated DEGs using the ConsensusClusterPlus package in R (Hu et al., 2022). Specifically, by using agglomerative pam clustering with person correlation distance after resampling 80% sample for 10 repetitions, we identified the optimal k-value and cluster number for further analysis (Hu et al., 2022). After identification of the optimal subgroup number, we first assessed the molecular function difference between subgroups via GSEA analysis in accordance with the MSIDB-sourced hallmark gene set using the clusterProfiler package in R (Wu et al., 2021). We also examined the expression patterns of KM-related DEGs between subgroups. Additionally, immune infiltration between subgroup was assessed using the CIBERSORT algorithm in R (Chen et al., 2018).

### 2.4 Machine learning analysis

After including normal and DMD samples in GSE6011 and GSE32720, we designated GSE6011 as the training set and GSE32720 as the independent validation set. We performed 12 machine learning models and 132 systemically combinations after combining these 12 machine learning models in the training set GSE6011 and validated in the independent validation set based on KM-related DEGs (Chen et al., 2024). In brief, the 132-model machine learning framework typically represents a comprehensive grid search approach that systematically combines multiple classification algorithms with various feature selection methods and preprocessing strategies (Chen et al., 2024). Combinations of these algorithms were applied to the MetaGSE for signature selection and model construction using 10-fold cross-validation (Chen et al., 2024). The optimal model was selected based on the highest mean area by achieving a high AUC-index (Chen et al., 2024). Next, model performance was examined by ROC and PR analyses using the pROC package in R (Robin et al., 2011). In addition, calibration and nomogram were performed in GSE6011 for examination model accuracy using the rms package in R (Lin et al., 2024). Indeed, SHAP analysis enabled the calculation of KM-related DEG contributions to model construction (Qi et al., 2025). After confirming the optimal contributor, we assessed the gene molecular and immune function using CIBERSORT and single-gene GSEA analysis using CIBERSORT and the clusterProfiler package in R (Chen et al., 2018; Wu et al., 2021). Additionally, the expression pattern of the optimal contributor was cross-assessed in GSE38417, GSE109178, GSE6011, and GSE32720.

### 2.5 Single-cell analysis

We acquired DMD mouse muscle tissue single-cell data (GSE213925) from the GEO database, which includes two DMD samples (Saleh et al., 2022). We first pre-processed this dataset (QC and reduction) using the Seurat package in R for the identification of reliable cell clusters (Satija et al., 2015). Cells with unique feature counts <200 or >6000 or with >15% mitochondrial gene content was filtered out (Butler et al., 2018). Data were normalized, and the top 2,000 highly variable genes were identified (Butler et al., 2018). PCA was followed by graph-based clustering and visualization with UMAP and t-SNE plots (Butler et al., 2018). Next, the singleR package in R enabled the identification of cell types, and markers for annotation are illustrated in Supplementary Table S5; (Cao et al., 2023). Cell communication patterns of cell types with ligand–receptor patterns were estimated using the Celltalker method in R (Wang et al., 2021). Metabolic patterns in these cell types were estimated using the SCENITH package in R (Argüello et al., 2020). In addition, AUCELL facilitated the identification of Kinic status among different cell types (Huang et al., 2024a). To investigate the functional impact of targeted molecules in targeted cells, we performed an *in silico* perturbation using the scTenifoldKnk R package (Osorio et al., 2022). scTenifoldKnk constructs a gene regulatory network (GRN) from single-cell RNA-seq data via tensor decomposition and simulates the removal of a target gene (Osorio et al., 2022). In brief, a k-nearest neighbor graph was constructed using the UMAP embeddings of targeted cells (n = X cells) (Osorio et al., 2022). A GRN was inferred for all expressed genes using the tenifold function with default parameters (Osorio et al., 2022). The target gene was then virtually knocked out by setting its expression to zero, and the perturbed GRN was recomputed (Osorio et al., 2022). Differential network analysis identified genes whose regulatory connections changed significantly after knockout, defined by a normalized knockout effect score >0.5 (the default threshold) (Osorio et al., 2022). The top 10 most perturbed genes (ranked by effect score) were subjected to GO and KEGG pathway enrichment, DO analysis, and GENEMINIA analysis using the clusterProfiler R package (FDR <0.05) (Xu et al., 2024; Chen et al., 2026). Finally, pseudotime trajectory analysis was conducted using the monocle2 package in R (Huang et al., 2024b).

### 2.6 Cell lines and culture

The human mast cell line (LAD2, ID:Eou84949) was purchased from Eoucell company (China). LAD2 maintained in high-glucose DMEM supplemented with 10% fetal bovine serum and 1% penicillin–streptomycin (Gibco, Germany). Furthermore, the LAD2 cell line deficient in exon 52 (DMDI) was generated by Sangon Biotechnology Company through the production of DMD-simulated LAD2 cell lines (Al et al., 2021). For the generation procedure, in brief, cells were transfected using two pSpCas9 (BB)-2 A-GFP plasmids, one containing a guide targeting the 5′ intron flanking exon 52 of the DMD gene and one containing a guide targeting the 3′ intron flanking exon 52 (Al et al., 2021). To generate the DMD cell line exhibiting a stop codon within exon 52 (DMDII), cells were transfected using a pSpCas9 (BB)-2 A-GFP containing a guide targeting exon 52 and a single stranded oligodeoxynucleotide (Integrated DNA Technologies) containing the mutated region (Al et al., 2021). Transfected GFP-positive single cells were cultured clonally (Al et al., 2021). For validation, screening was performed by PCR using primers flanking the deleted region, and clones exhibiting perfect repair by nonhomologous end joining were selected after sequencing (Al et al., 2021).

### 2.7 qRT-PCR

Total RNA was extracted using TRIzol reagent (TaKaRa, China). For cDNA synthesis, the HiScript II Q RT SuperMix (Vazyme) was used. qRT-PCR assays were performed in triplicate using a StepOne instrument (Applied Biosystems, USA) with SYBR Green MasterMix (YEASEN, China), with 40 amplification cycles per run. Relative gene expression levels were determined using the ΔΔCt method, normalizing GAPDH as an internal control. The sequences of the primers used are listed in Table 2 (Wang et al., 2025). This study was performed with three biological replicates.

**TABLE 2:** Primers used in this study.

Name	Primers for PCR (5′–3′)
USP18	Forward: CATGGCGCTTGAGAGATTCC Reverse: CAACCAGGCCATGAGGGTAG
GAPDH	Forward: GGAGCGAGATCCCTCCAAAAT Reverse: GGCTGTTGTCATACTTCTCATGG

### 2.8 Western blotting

Cells were washed with ice-cold PBS and harvested via gentle scraping. Protein isolation was carried out using RIPA buffer supplemented with phosphatase and protease inhibitors. The lysates underwent centrifugation at 14,000 × g for 15 min at 4 °C, and the resulting supernatants were denatured with 5× SDS-PAGE loading buffer. After separation through SDS-PAGE, proteins were transferred onto PVDF membranes and blocked with NcmBlot blocking buffer. For primary antibody incubation, the membranes were maintained at 4 °C overnight with anti-USP18 (ABCAM; dilution 1:1,000) and anti-GAPDH (ABCAM; dilution 1:2000), both prepared in 5% BSA. Post-washing, the membranes were exposed to an HRP-conjugated secondary antibody (dilution 1:1,000) for 2 h at room temperature.

### 2.9 Drug acquisition

To systematically identify potential therapeutic compounds targeting DMD, we utilized the DrugReflector platform in GSE6011 (43). In brief, Drugreflector is a multi-omics drug repositioning database integrating transcriptomic, proteomic, and protein–protein interaction data (DeMeo et al., 2025). GSE6011 was input as the sole target. Each compound was scored using the Drug–Target Association Score (DTAS), which combines gene expression similarity, chemical structure similarity, known drug–target interactions, and network topological features (DeMeo et al., 2025). All candidates were ranked in descending order of DTAS. The initial shortlist included compounds with DTAS >0.9 and ranking within the top 10 (43). In addition, the *in silico* pharmacology network based on disturbed molecules caused by KO of the optimal indicator interaction pattern was used for the identification of the therapeutic agent targeting the hub contributor (Guney et al., 2016). Moreover, the binding affinity between the contributor and the agent was assessed using molecular docking (Zhou et al., 2026). The protein structures (acquired from the alphafold2 database) were initially prepared using PyMOL software (Zhou et al., 2026). AutoDock Vina was used to predict potential binding sites on the protein surface and to execute flexible docking simulations (Zhou et al., 2026). This process yielded docking scores and binding affinities (kcal/mol) for each identified cavity, and the conformation with the most favorable energy was selected for further analysis (Zhou et al., 2026). The three-dimensional (3D) binding mode, including specific hydrogen bond interactions, was visualized using PyMOL (8). Additionally, two-dimensional (2D) diagrams detailing the molecular interactions, such as hydrogen bonds, hydrophobic contacts, and π–π stacking between amino acid residues and the compounds, were generated using MOE software (Zhou et al., 2026).

### 2.10 Statistical analysis

Statistical analyses were performed using R software (Version 4.2.2) and GraphPad Prism software (Version 9.2.2). Comparisons between two groups were performed using Student’s t-test, while comparisons among multiple groups were conducted using one-way ANOVA. A threshold of p < 0.05 and a false discovery rate (FDR) below 0.05 were set to define statistical significance.

## 3 Results

### 3.1 Identification of the gene module highly correlated with Kinic in DMD

In GSE38417, we identified that Kinic was restrained in DMD patients compared to normal controls (Figure 1D). Next, WGCNA analysis identified that the black module was most highly correlated with Kinic (R = 0.49) and DMD pathogenesis (R = −0.86) in GSE38417, so we extracted genes in this module and designated them as Kinic-correlated genes (Figures 1A–C,E–G).

**FIGURE 1:**
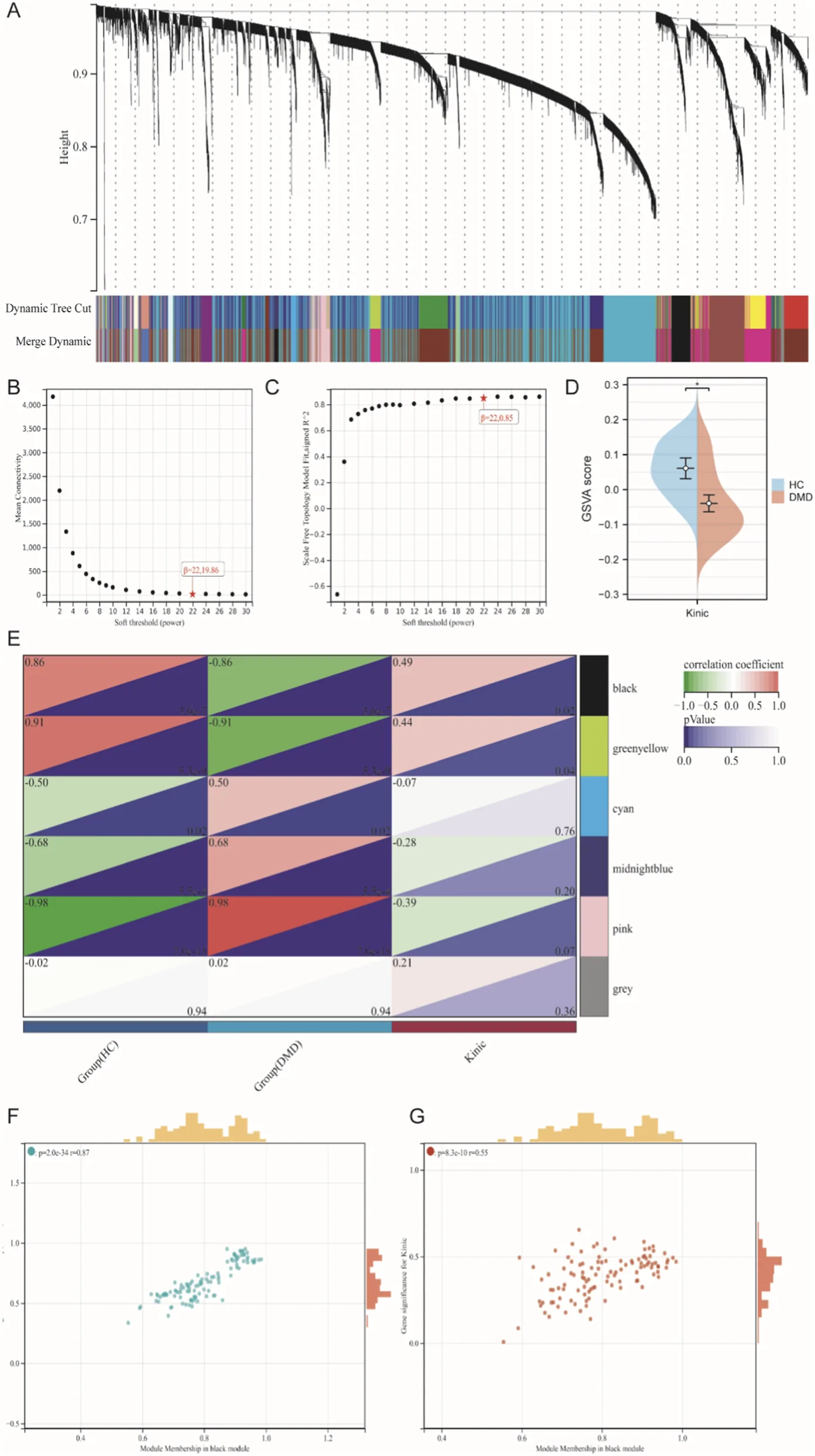
Identification of Kinic-correlated genes for DMD patients. **(A)** WGCNA dynamic cutting tree in GSE38417 for module confirmation. **(B,C)** β threshold of WGCNA analysis for module confirmation. **(D)** GSVA enrichment of the Kinic score in GSE38417. **(E)** Correlated heatmap of each module in WGCNA analysis. **(F,G)** Black module-correlated map for DMD and Kinic scores in GSE38417.

### 3.2 Identification of the gene module highly correlated with mast cells in DMD

In GSE109178, we discovered that mast cell activation was downregulated compared to the normal control (Figure 2B). Next, WGCNA analysis identified that the green module was most highly correlated with mast cells (R = −0.63) and DMD pathogenesis (R = 0.91) in GSE10178, so we extracted genes in this module and designated them as mast cell-correlated genes (Figures 2A,C–G).

**FIGURE 2:**
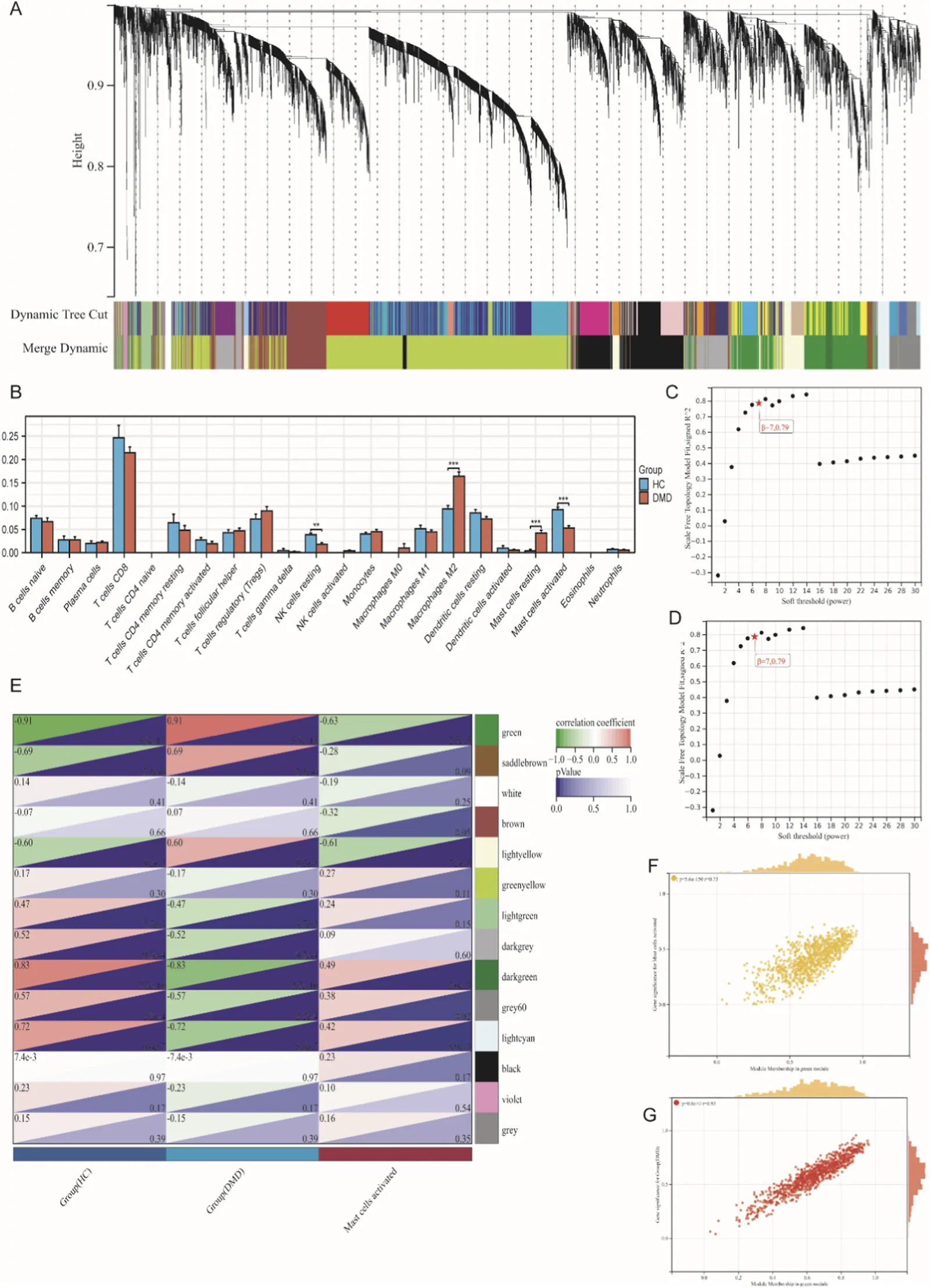
Identification of mast cell-correlated genes for DMD patients. **(A)** WGCNA dynamic cutting tree in GSE109178 for module confirmation. **(B)** CIBERSORT enrichment immune cell score in GSE109178. **(C,D)** β threshold of WGCNA analysis for module confirmation. **(E)** Correlated heatmap with mast cells of WGCNA analysis **(F,G)** Black module-correlated map for DMD and mast cell scores.

### 3.3 Identification of KM-related molecular subgroups in DMD

After intersecting Kinic- and mast cell-correlated genes, we identified nine shared KM-associated DEGs and discovered that these DEGs were mainly involved in maintaining intracellular homeostasis (Figures 3A–D; Supplementary Tables S2–S4). Next, based on these nine DEGs, in GSE6011, we divided DMD patients into two groups (C1 and C2) (Figures 3E–G). Next, DEG expression between these two groups was estimated, and molecular and immune characteristics between these two groups were also assessed (Figures 3H–J).

**FIGURE 3:**
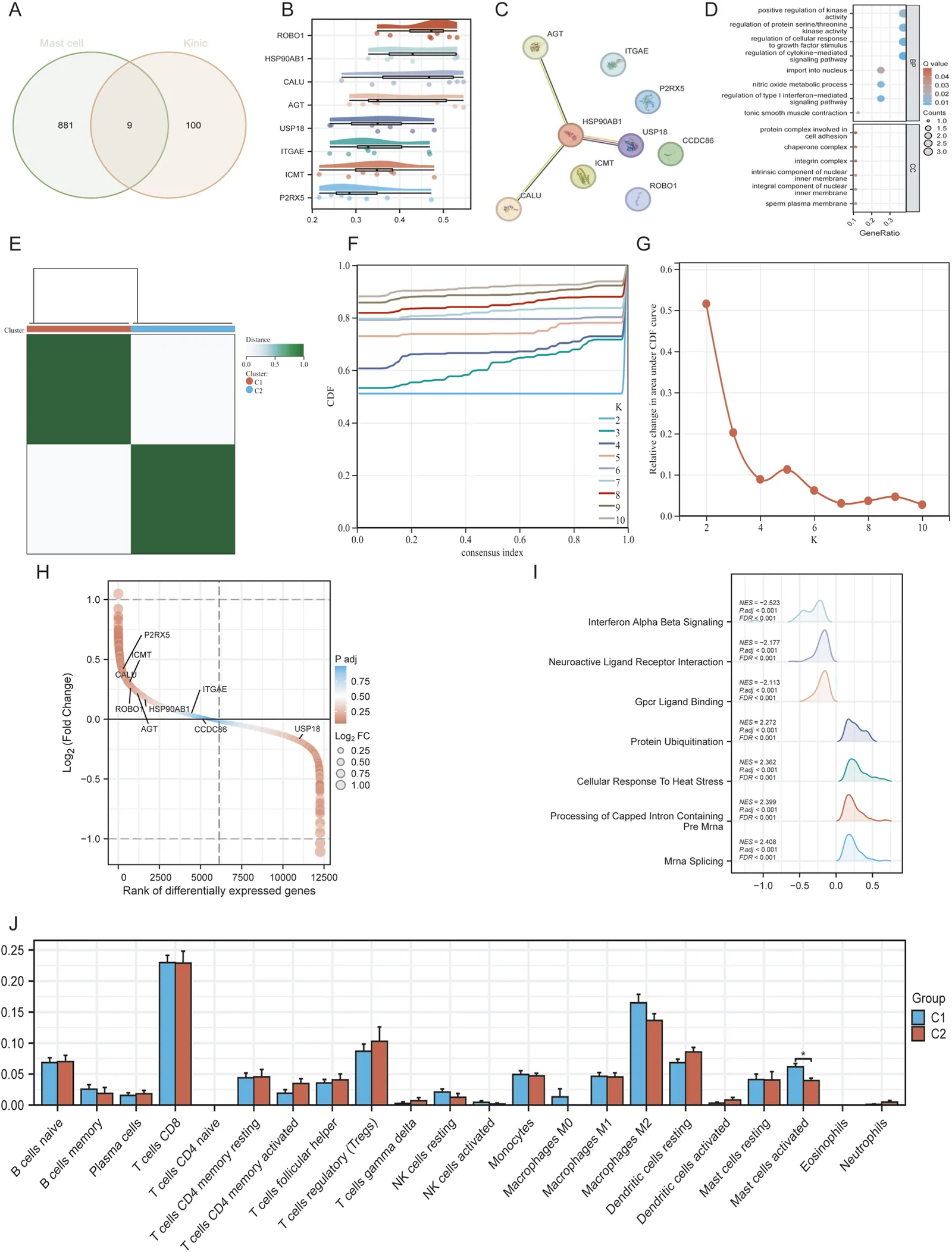
Identification of KM-related subgroups for DMD patients. **(A–D)** Identification of KM-associated DEGs and corresponding molecular pattern estimations. **(E–G)** Consensus clustering for identification of KM-related subgroups. **(H)** Expression patterns of KM-related DEGs between subgroups. **(I)** GSEA analysis between these two groups. **(J)** Immune infiltration analysis between these two groups.

### 3.4 KM-related diagnostic model construction in DMD

Using 132 systemically machine learning models in GSE6011 and GSE32720, we identified that RF + Stepglm can be considered the optimal choice for construction of the diagnostic model based on KM-related DEGs (Figure 4A). In addition, in GSE6011 and GSE32720, the model illustrated favorable performance (Figures 4B–E). Next, we examined the model efficacy using nomogram and calibration analysis (Figures 4F–H).

**FIGURE 4:**
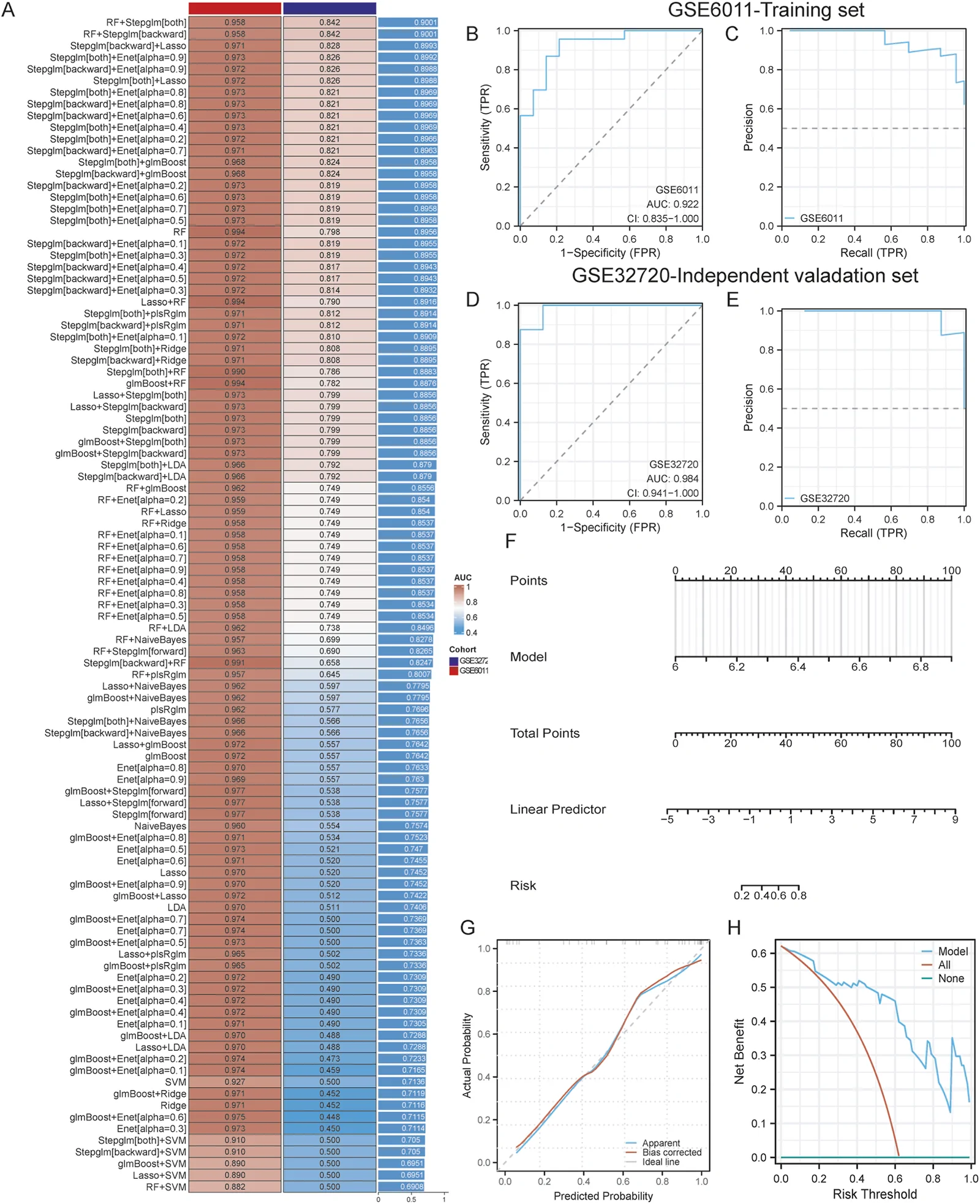
KM-related diagnostic model construction in DMD. **(A)** Construction of 132 systemically machine learning models for DMD patients. **(B–E)** ROC and PR examination of model performance in GSE6011 and GSE32720. **(F–H)** Nomogram and calibration model examination in GSE6011.

### 3.5 KM-related hub gene identification in DMD

According to the SHAP results, we discovered that USP18 can be considered the optimal diagnostic candidate for model construction among KM-related DEGs (Figure 5A). Next, we also discovered that USP18 expression was upregulated in DMD samples compared to normal controls across GSE38417, GSE32720, GSE 6011, and GSE32720 (Figures 5C–F). Next, we estimated USP18 molecular functions in GSE6011 and discovered that USP18 was mainly involved in signals related to inflammation (Figure 5B). *In vitro* assays also validated the expression of USP18 patterns (Figures 5G,H). Next, we also discovered that SUP18 high-expression was negatively related to mast cell activation (Figure 5I).

**FIGURE 5:**
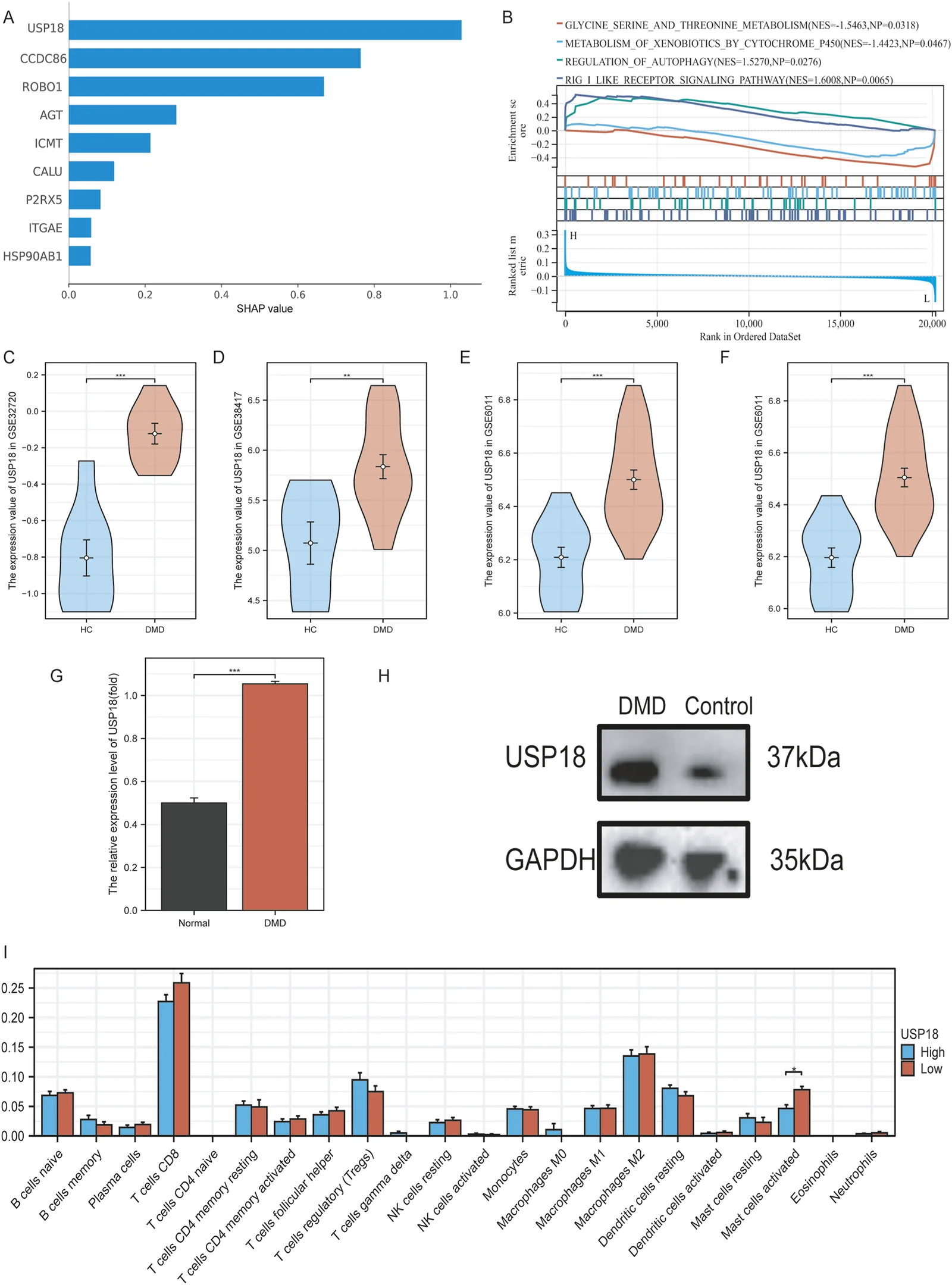
Identification of KM-associated hub genes in DMD. **(A)** SHAP analysis of contributors in the machine learning model. **(B)** Single-gene GSEA analysis of USP18 in GSE6011. **(C,F)** *In silico* examination of USP18 expression. **(G,H)** *In vitro* examination of USP18 expression. **(I)** Immune infiltration analysis of USP18 in GSE6011.

### 3.6 DMD single-cell atlas illustration

In GSE213925, we identified 382,392 cells, 24 cell clusters, and 10 cell types after pre-processing (Supplementary Figure S1D; Figure 6A; Figure 6B; Figure 6C; Supplementary Figure S1A). Mast cells exhibited a relatively low proportion among these 10 cell types (Figure 6D). In addition, cell–cell communication and metabolic characteristics of these 10 cell types were estimated (Figures 6E,F).

**FIGURE 6:**
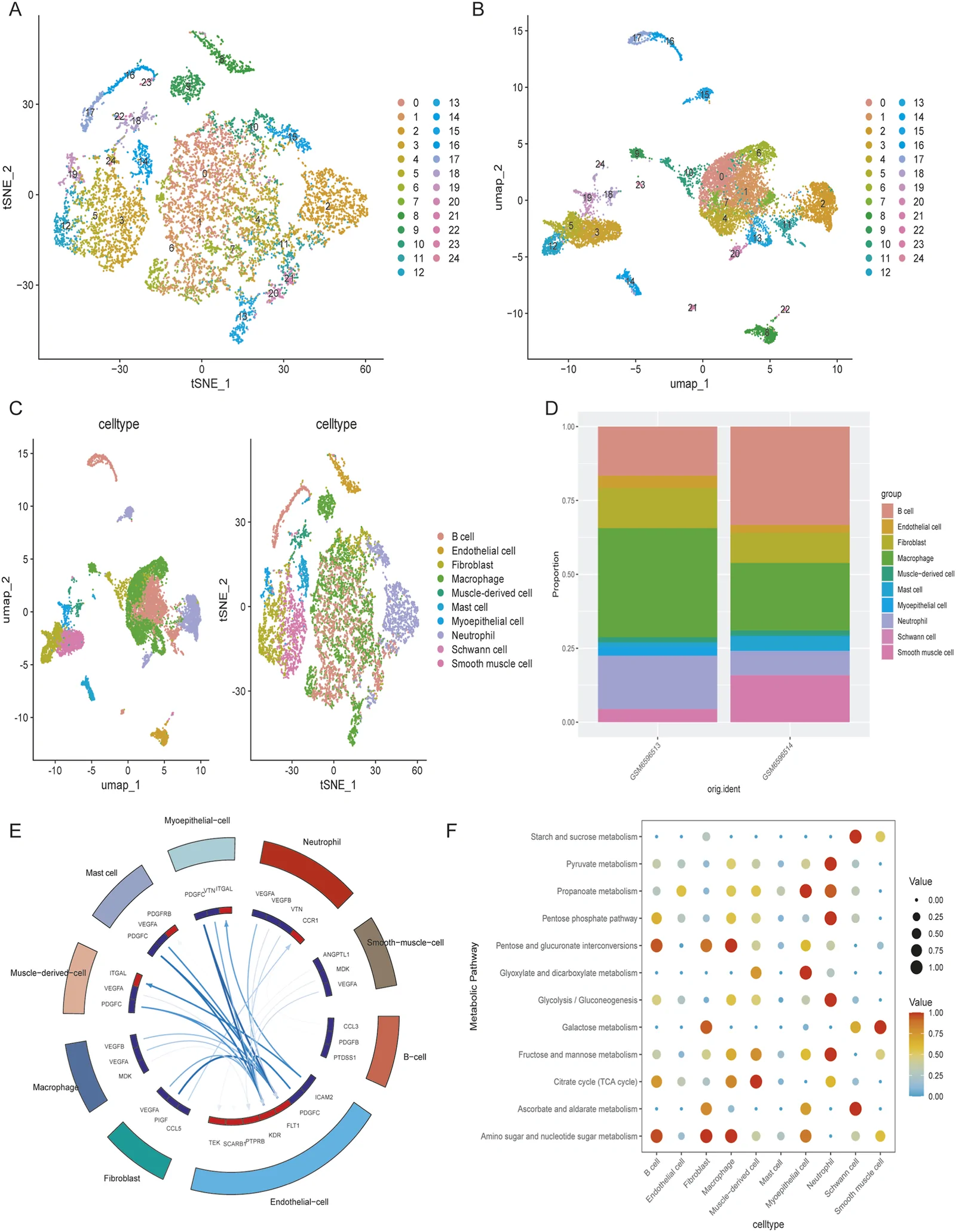
DMD single-cell atlas illustration. **(A,B)** Cell cluster identification in GSE213925. **(C)** Cell type identification in GSE213925. **(D)** Proportion of cell types in GSE213925. **(E)** Cell–cell communication analysis among various cell types. **(F)** Metabolic patterns in different cell types.

### 3.7 KM-associated hub gene mast cell-regulated mechanisms in DMD

AUCELL identified that Kinic was relatively activated in mast cells and that Usp18 was distributed in mast cells (Figures 7A,B). After virtual KO of Usp18 in mast cells, we identified that perturbed USP18 may potentially affect molecules involved in the maintenance of muscular functions and related to muscular disease (Figures 7C–F). In addition, we also identified that there are five trajectories and that Usp18 expression was correlated with mast cell differentiation (Figures 7G,H).

**FIGURE 7:**
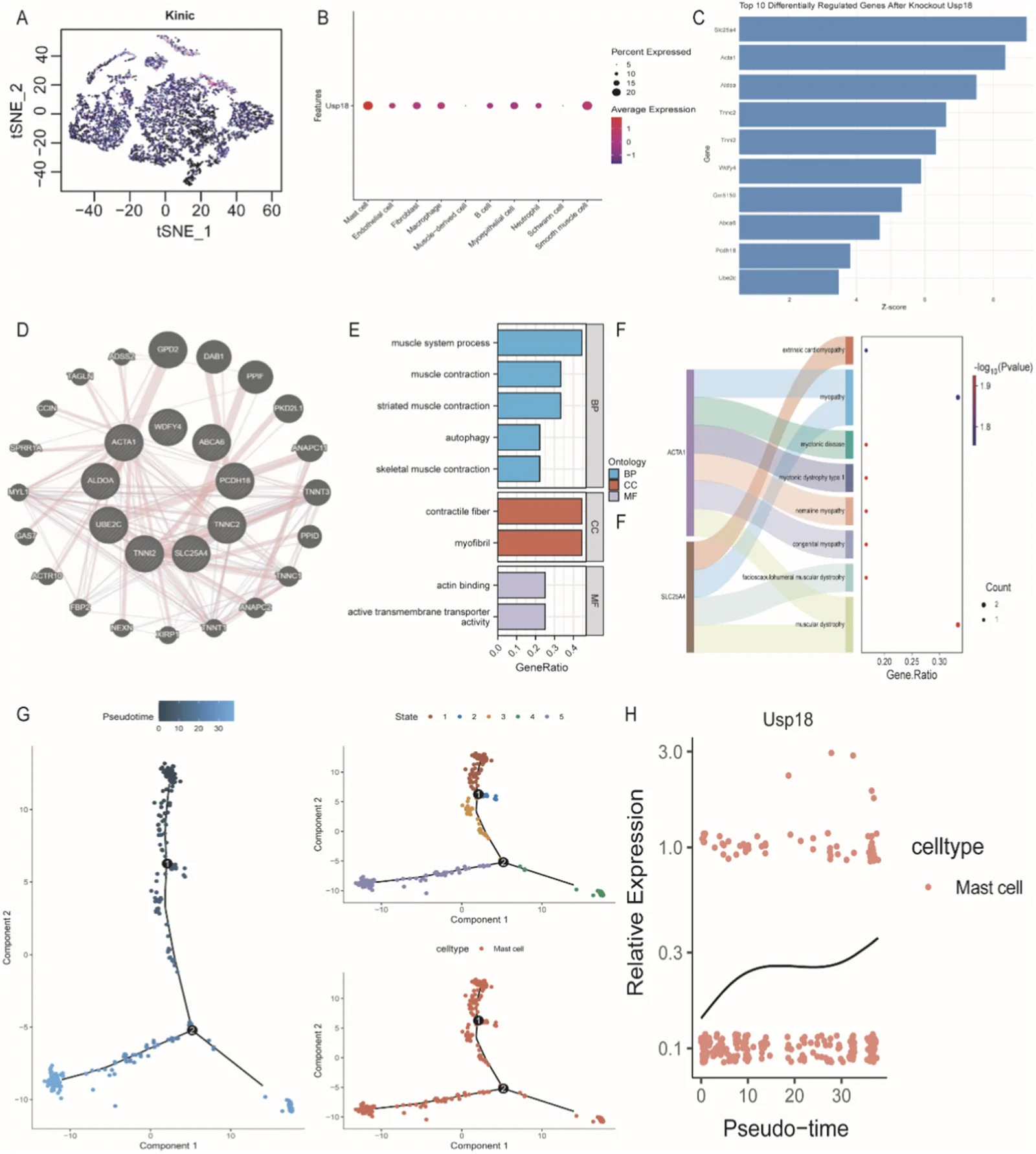
Usp18 molecular mechanisms in DMD mast cells. **(A)** AUCELL analysis of Kinic activity. **(B)** Cell distribution of Usp18 among annotated cell types. **(C–F)** KO of Usp18 patterns in mast cells in the DMD mouse model. **(G,H)** Monocle2 analysis of mast cells and Usp18 expression in mast cell differentiation.

### 3.8 Drug screening for treatment of DMD

In GSE6011, we first identified agents for the treatment of DMD using the deep learning pipeline and discovered that BRD-K10482608 was the optimal choice (Figure 8A). Next, based on the disturbed molecular patterns of USP18 in mast cells, we discovered that Finafloxacin can be considered a USP18-targeted agent for the treatment of DMD with satisfied binding affinity (Figures 8B–D).

**FIGURE 8:**
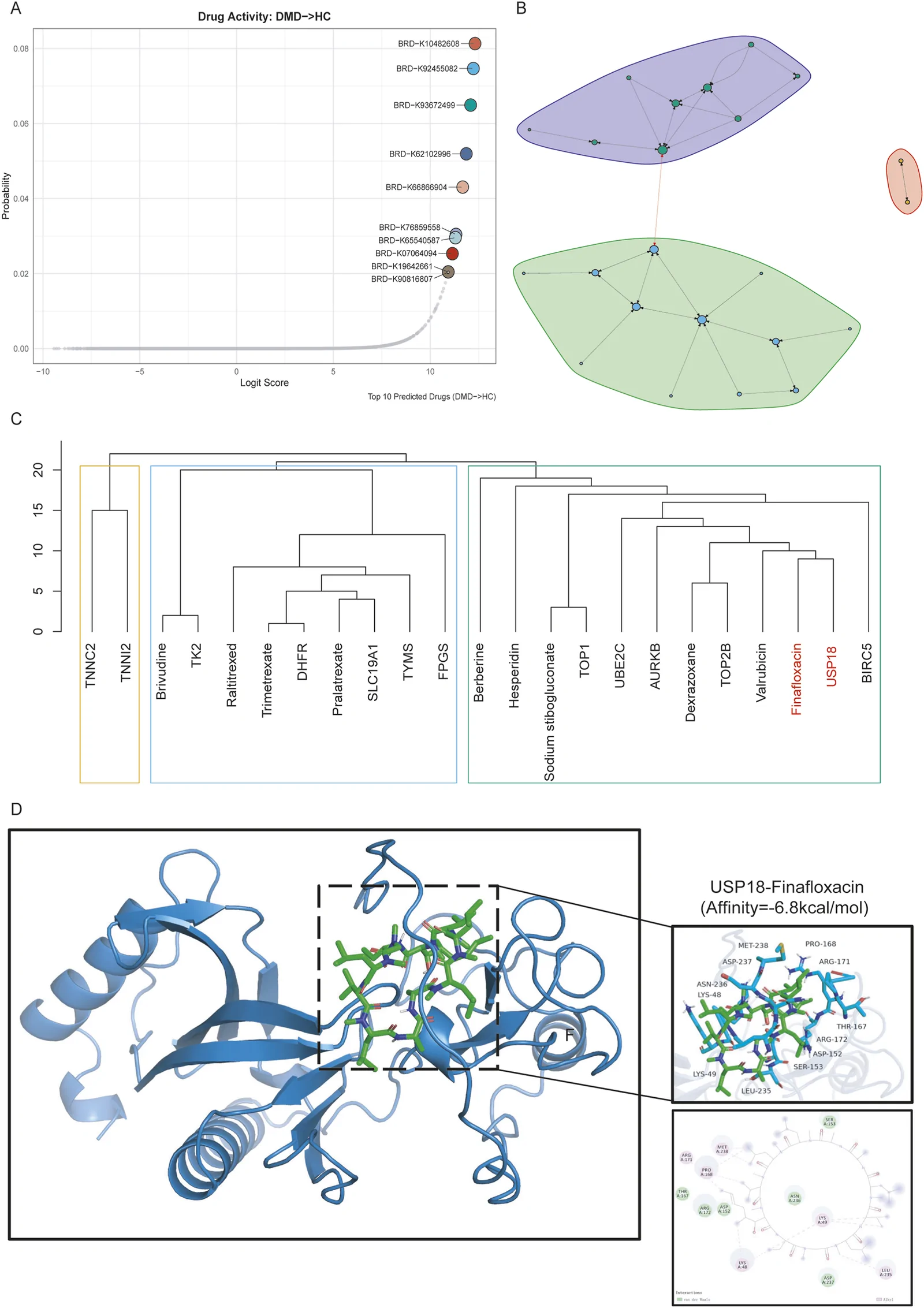
Drug enrichment for DMD treatment. **(A)** Deep learning framework estimation in GSE6011. **(B,C)** Network pharmacology enrichment of the USP18-targeted drug. **(D)** Molecular docking assessment.

## 4 Discussion and conclusion

DMD was an X-linked neuro-muscular disease posing threat on children and young adults (Yiu and Kornberg, 2015). However, current diagnostic and therapeutic strategies were mainly focused on and based on symptom discovery and addressing symptoms because of disease heterogeneity (Tang and Yokota, 2024). Therefore, efficient predictive and therapeutic approaches should be developed to increase precision and personalized medicine for DMD patients.

USP18, an enzyme responsible for ubiquitination, has widely been reported in muscular disorder (Hou et al., 2021). In DMD, for example, USP18 can be considered an ISG15 activator for promoting inflammation in dermatomyositis muscle with perifascicular atrophy and juvenile inflammatory myopathies (Salajegheh et al., 2010). However, previous reports have not yet elucidated the pathogenic role of USP18 in DMD. In addition, USP18 can be considered a non-histone target for Kinic modification (Li et al., 2024). In regulation of mast cell, USP18 was identified as the susceptible molecule associated with DMD pathogenesis (Granchelli et al., 1995). Nevertheless, a knowledge gap remains between USP18 and mast-cell-integrated mechanisms involved in DMD pathogenesis, which hindered the understanding of DMD pathophysiology.

In our study, we bridged the gap between Kinic modification and mast-cell-integrated gene signature involved in DMD pathogenesis, which enriched the knowledge in DMD pathogenesis. In addition, we suggest a KM-related predictive and therapeutic model for DMD patients, which enhanced DMD patient clinical translation. Finally, we first identified USP18 and estimated its pathogenic role in DMD. Nevertheless, there are still limitations in our study that should be addressed in the future. This study inferred Kinic-associated activity from a transcriptomic gene signature, rather than direct mass-spectrometry or antibody-based measurement of Kinic; the Kinic-related findings should be interpreted as signature-based associations. Future studies should focus on decoding the Kinic catalyzation patterns in DMD pathogenesis. Moreover, we only quantified the expression of the Kinic-associated central pathogenic factor (USP18) at *in vitro* assays; the Kinic-modified molecular mechanisms of USP18 in DMD pathogenesis should be determined in future pre-clinical studies. In addition, the foundational transcriptomic insights are derived from publicly available datasets, which, despite batch correction, may not fully account for all sources of technical and biological variability. Future studies should combine the clinical information of DMD patients, focusing on the estimation of efficacy of diagnostic and molecular stratification models acquired from our study in a large, multi-center clinical trial to enhance clinical significance. The single-cell analysis, while offering high-resolution insights, is based on a cohort including two DMD mouse models, potentially limiting the capture of the full heterogeneity of mast cell states across the DMD spectrum. Future studies should combine advanced AI technologies with higher-resolution single-cell and spatial data from DMD patients, coupled with preclinical studies, dynamically tracing Kinic mechanisms in mast cells involved in the pathogenesis of DMD. Similarly, the therapeutic candidates identified computationally in our study, such as BRD-K10482608 and finafloxacin, demand rigorous experimental evaluation in preclinical models to confirm its efficacy, optimal dosing, and ability to penetrate the muscle tissue and modulate the proposed Kinic in mast cells and USP18 Kinic levels to enhance their clinical translational potential.

## 5 Statements and declarations

### 5.1 Mandatory AI declaration

All authors confirm that no AI tools (including but not limited to large language models, AI writing assistants, AI data analysis software, or AI editing tools) were used in any stage of manuscript creation. This includes, but is not limited to, conceptualization of the study, design of methodology, data collection and analysis, drafting of the manuscript, critical revision of intellectual content, editing for grammar or structure, and preparation of figures/tables. All work presented in the manuscript is the original intellectual product of the authors, with contributions directly made by each author as outlined in the Author Contributions Statement.

## Data Availability

The bulk RNA-seq and single-cell RNA-seq data of Duchenne muscular dystrophy (DMD) analyzed in this study are all publicly available from the Gene Expression Omnibus (GEO) database (https://www.ncbi.nlm.nih.gov/geo/) under the accession numbers GSE38417, GSE109178, GSE6011, GSE32720, and GSE213925. The isonicotinylation (Kinic)-associated gene list used in the study was obtained from the published peer-reviewed literature. All original data generated from *in vitro* experiments (qRT-PCR and Western blotting) in this study are available from the corresponding author on reasonable request. The R code and related analytical pipelines applied for bioinformatic analysis can be provided by the corresponding author upon reasonable request.

## Supplementary material

SUPPLEMENTARY FIGURE S1 Pre-processing of single-cell data. **(A–C)** QC metrics of single-cell data. **(D)** Marker of each cell cluster.
